# Three-dimensional bio-printing and bone tissue engineering: technical innovations and potential applications in maxillofacial reconstructive surgery

**DOI:** 10.1186/s40902-020-00263-6

**Published:** 2020-06-03

**Authors:** Muhja Salah, Lobat Tayebi, Keyvan Moharamzadeh, Farhad B. Naini

**Affiliations:** 1 St George’s Hospital, London, UK; 2 Marquette University School of Dentistry, Milwaukee, WI USA; 3 Academic Unit of Restorative Dentistry, School of Clinical Dentistry, University of Sheffield, Sheffield, UK; 4 Kingston and St George’s Hospitals and St George’s Medical School, London, SW17 0QT UK

**Keywords:** Facial reconstruction, Bone graft, Bone tissue engineering, Additive manufacturing, Bioactive scaffolds, 3D bioprinting, Bioink

## Abstract

**Background:**

Bone grafting has been considered the gold standard for hard tissue reconstructive surgery and is widely used for large mandibular defect reconstruction. However, the midface encompasses delicate structures that are surrounded by a complex bone architecture, which makes bone grafting using traditional methods very challenging. Three-dimensional (3D) bioprinting is a developing technology that is derived from the evolution of additive manufacturing. It enables precise development of a scaffold from different available biomaterials that mimic the shape, size, and dimension of a defect without relying only on the surgeon’s skills and capabilities, and subsequently, may enhance surgical outcomes and, in turn, patient satisfaction and quality of life.

**Review:**

This review summarizes different biomaterial classes that can be used in 3D bioprinters as bioinks to fabricate bone scaffolds, including polymers, bioceramics, and composites. It also describes the advantages and limitations of the three currently used 3D bioprinting technologies: inkjet bioprinting, micro-extrusion, and laser-assisted bioprinting.

**Conclusions:**

Although 3D bioprinting technology is still in its infancy and requires further development and optimization both in biomaterials and techniques, it offers great promise and potential for facial reconstruction with improved outcome.

## Background

Developmental or acquired craniofacial deformities may impose a negative psychological impact on affected individuals [1]. Such deformities affect the soft and the hard tissues of the craniofacial complex to varying extents. Managing such patients requires multidisciplinary care, with the patient under the care of a team of specialists that may involve a cranio-maxillofacial surgeon, plastic and reconstructive surgeon, otorhinolaryngological surgeon, neurosurgeon, pediatrician, orthodontist, psychologist and speech and language therapist. Combined specialists’ effort is needed to improve function and aesthetics.

Surgery and bone grafting are considered crucial pillars for repair when missing large amounts of bone [2]. Bone grafts may be harvested from the patient as an autologous graft, which has the advantage of being biocompatible and not eliciting an immune response or provoking graft rejection. Alternatively, allogenic bone can be harvested from cadavers and transplanted into recipients, which carries the risk of rejection. Bone can also be replaced using natural or synthetic bone substitutes [3]. Grafts provide support to the soft tissue overlaying it, improving the aesthetics and allowing for the healing process to occur by “creeping substitution,” which is the resorption of graft and its replacement with new bone [4]. This is accomplished by the ability of the graft to osteoinduce and osteoconduct the healing process. Osteoinduction entails stimulation of progenitor cells to differentiate into osteoblasts and enhance bone deposition, while osteoconduction means providing a surface on which the new bone will be deposited to direct the bone growth and restore its shape [5, 6].

For the bone graft to be successful, it should match the shape and size of the replaced bone, in order to offer the support and conduct the new bone formation [7]. Craniofacial bones have a complex architecture that encompasses spaces and contains delicate organs, and shaping a bone graft in the traditional way with bone trimming requires burs that will not allow replication of the exact facial bone intended to be replaced [8]. Transplanting a graft that does not mimic the original shape of the bone will negatively affect the outcome of the facial reconstructive surgery. Therefore, developing an appropriate scaffold material to replace bone along with the appropriate technique has been the focus of ongoing research in the last decade.

Bone tissue engineering has been an avenue of scientific interest since the 1980s [9, 10]. It aims to fabricate substitutes for bone grafts using a scaffold from a biocompatible material that carries the osteogenic cells needed for healing along with the growth factors that will help the osteodifferentiation and angiogenesis to aid in the healing process [11]. Since then, many biomaterials have been developed and tested to fulfill the requirement needed for safety, adequate mechanical properties and designs to mimic the replaced bone [12, 13].

Many methods have been utilized to fabricate scaffolds for tissue engineering, including solvent casting and particulate leaching (SCPL), phase separation, gas foaming, microsphere sintering and electrospinning [14, 15]. Though these methods are able to produce a scaffold out of biomaterial, they are unable to precisely depict the design they are intended to replace. Fortunately, the collaboration between scientists and engineers, together with interdisciplinary research, led to significant developments. Early in the 1970s, advances in design and manufacturing technology led to the development of the CAD/CAM system (computer-aided design/computer-aided manufacturing), which was adopted and implemented heavily in the automotive and aerospace industries [16]. The system produces a digital design using software, and then with the assistance of the computer, a prototype is manufactured. The technology made its way into dentistry where it is still utilized for the fabrication of various fixed partial dentures and restorations requiring better aesthetic appearance and durability [17]. Manufacturing those prototypes depends on carving the design from an ingot or a block of material using the CAM system [18, 19]. Scaffolds, on the other hand, require having interconnected pores to ensure infiltration with nutrients and ensure cell migration within the material to allow for replacement with healing cells [20].

Advances in manufacturing continued, and additive manufacturing (AM) or three-dimensional (3D) printing, was introduced into industry and biology. The AM technology fabricates the prototype or scaffold by depositing bioink in a layer by layer manner rather than subtracting from an ingot [21, 22]. Bioink is a material that resembles the extracellular matrix containing cells encapsulated within, which is then deposited with precision and polymerizes or cross-links either before or after deposition to form the scaffold [23]. Previously, the technology was used to deposit a single bioink but nowadays and with the advancement of the field, multicomponent bioinks may now be deposited in high accuracy to mimic the complex architecture of human tissue [24]. Recently, the CAD technology combined with the advances in the AM and 3D bioprinting technology has succeeded in the construction of scaffolds for tissue regeneration that was translated and implanted in patients for the repair of the blood vessels, urethra, urinary bladder, and trachea [25].

The present review introduces both the bioink material and the technology used in 3D bioprinting in relation to maxillofacial plastic and reconstructive surgery.

## Bioink material

The first generation of bioinks are materials that show bioinert behavior and biocompatibility, which do not elicit an immune response or rejection. In contrast, the body only responds by forming a thick fiber capsule; thus, the scaffold remains implanted without degrading and continues to provide mechanical support, e.g. metals (stainless steel and titanium) and polymers (silicone and polymethylmethacrylate) [26]. The second generation of bioinks are materials demonstrating both biocompatibility and being bioactive. They allow mineralization, i.e. hydroxyapatite formation and are able to be biodegradable for healing cells to replace the scaffold [27]. The third generation of bioinks is the bioresponsive materials, which incorporate growth factors and stimulatory molecules that stimulate osteoblast differentiation including bone morphogenetic proteins (BMP) and fibroblast growth factors (FGF) [28]. Nowadays, a multicomponent bioink is used, as it combines the mechanical and functional properties to meet the complex need within the replaced tissue [24].

## Biomaterials used for scaffold construction

### Polymers

Polymers are an organic material made of repeated subunits, called monomers, bonded together with covalent bonds and composed mainly of carbon atoms [29]. They are a versatile material that has shown to be both biocompatible and biodegradable, with the ability to change its mechanical properties by altering its chemical structure. It can be highly viscous, such as hydrogel polymer, or it can be stiffer to produce a stronger scaffold, such as polycaprolactone (PCL) [30]. Yet, the mechanical properties of the polymer depend on the tissue it is replacing and the method of 3D printing [31]. If inkjet printing is used, stiff bioink can clog the nozzle of the printer as the ink is deposited. This could be managed by depositing unpolymerized ink that solidifies and cross-links after deposition.

Polymer materials are either natural or synthetic. Natural polymers include proteins (e.g. silk, gelatin and collagen) and polysaccharides (e.g. alginate, agarose and chitosan). Polysaccharide polymers provide a lower antigenicity, but also have lesser mechanical properties than protein polymers [32]. On the other hand, polyhydroxy acids are synthetic polymers, such as polylactic acid (PLA), polyglycolic acid (PGA) and polylactic-glycolic acid (PLGA) [33, 34]. They are successful in producing a porous scaffold, but biodegrade after implantation to produce lactic acid and carbon dioxide. These byproducts can be easily removed from the body; however, they simultaneously produce an acidic environment that favors inflammation instead of healing [35]. Polycaprolactone (PCL) is a synthetic polymer that is found to biodegrade safely by hydrolysis degradation, thus not negatively affecting the tissue environment, unlike the polyhydroxy acid polymers [36]. It also has a low melting point, making it compatible with encapsulating live cells while being deposited without affecting their viability.

### Bioceramics

Ceramics are inorganic materials in the form of bioactive glass (BG), metal oxides or bioactive ceramics developed for medical and dental use to replace bone [37]. BGs are composed of silicon dioxide or silicate with sodium dioxide, calcium oxide and phosphorus [38]. They induce hydroxyapatite (Hap) formation after contacting biological fluid, thus enhancing osteogenesis and bone healing [39]. On the other hand, bioactive glass ceramics (e.g. Hap and tricalcium phosphate) are materials that bond directly with bone without the formation of an intermediate fibrous connective tissue layer [40]. Bioceramic materials are brittle, have a low mechanical strength and low fracture toughness and thus cannot be used solely for scaffold fabrication [6].

### Composites

A composite material is a mixture of two or more different materials with the intent to manipulate the mechanical properties of the end material utilizing the properties of the initial materials used [41]. The composite is formed of polymer mixtures or polymer-ceramic mixture. For example, mixing PCL with Hap enhances the brittleness of the Hap and decreases the hydrophobicity of the PCL, thus increasing cell attachment and cell infiltration into the scaffold [42].

## Stem cells and bioactive molecules

For the biomaterial to be a bioink, they should encapsulate stem cells that can differentiate into different lineages and assist in the body healing and regeneration [43]. Stem cells may be embryonic (ESC) in origin, which carry the ethical considerations of harvesting them using embryos [44]. Alternatively, adult stem cells may be found in specific niches within the adult body, such as the bone marrow, umbilical cord, amniotic tissue, dental pulp and adipose tissue. Shen et al. compared the osteogenic differentiation potential of different mesenchymal stem cell (MSC) sources and determined that MSC derived from the amniotic membrane and umbilical cord tissue has the highest osteogenic potential compared with other MSC-derived cells [45]. Bioactive molecules incorporated within the bioink are growth factors that enhance angiogenesis, as in vascular endothelial growth factor (VEGF), or may also improve osteogenic differentiation and bone formation, as in BMP, FGF and insulin-like growth factor-1 (IGF-1) [23].

## Bioprinting process

**Fig. 1:**
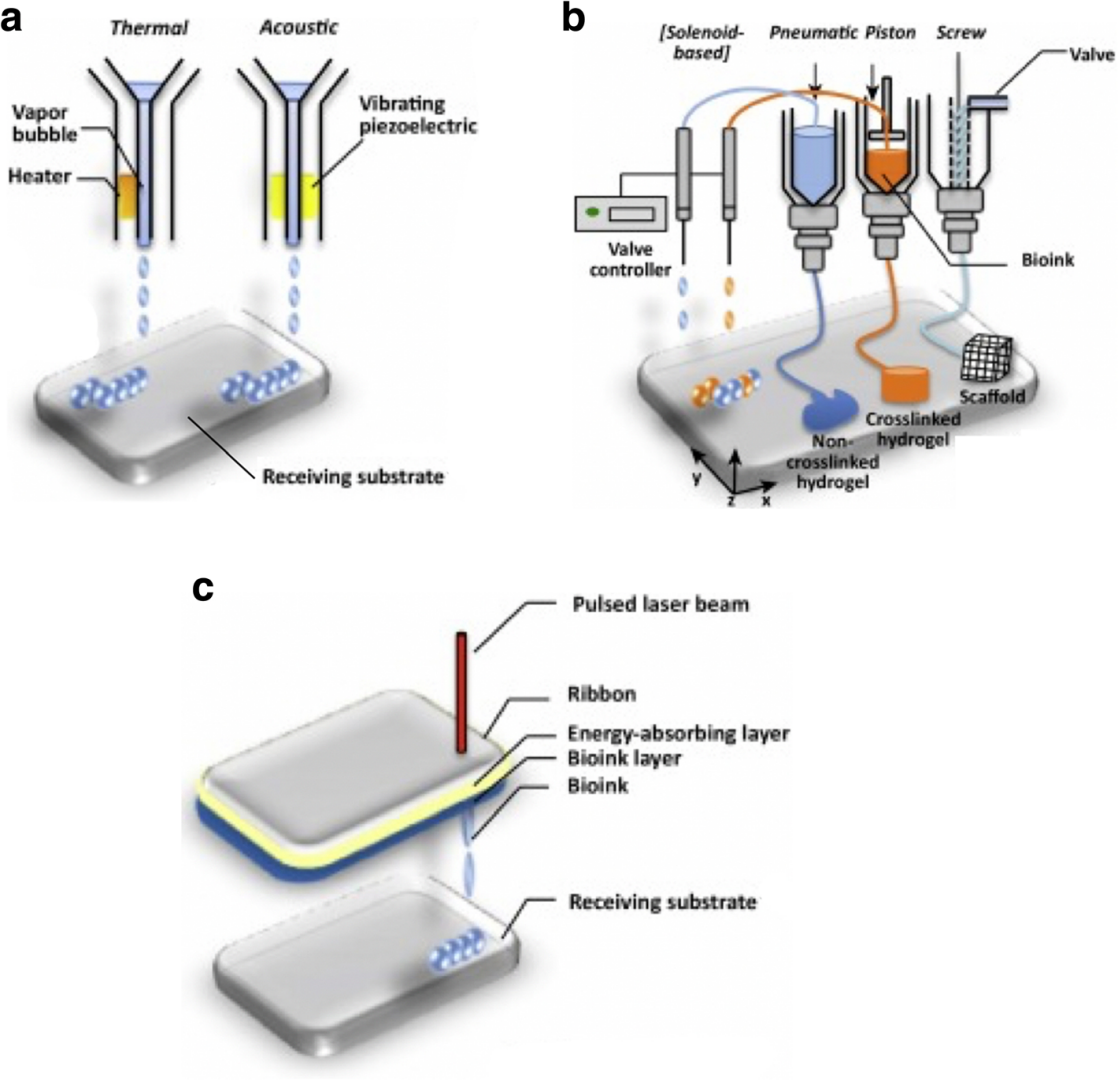
Schematic representation of the 3D bioprinting techniques: **a** inkjet bioprinting, **b** micro-extrusion bioprinting, and **c** laser-assisted bioprinting (modified and adapted from Visscher et al.) [47]

Bioprinting is an additive manufacturing (AM) process in which the bioink, containing the ECM-like material with encapsulated stem cells and active molecules, is deposited in the precise location with the aid of the CAD system to form the scaffold. This facilitates the restoration of the complex micro-environment of the native tissue to enhance healing by cell differentiation, which is called biomimicry [46]. Different modalities are used in the 3D bioprinting to fabricate the scaffold, which are the following (Fig. 1) [48].

### Inkjet bioprinting (or drop-on-demand bioprinting)

The bioink in this printer is kept in a chamber above the nozzle where it is subjected to a change in pressure created by heating the bioink, which subsequently leads to the ejection of a drop of the bioink into a platform beneath the nozzle. The high heat required to create this pressure affects cell viability [49]. Otherwise, the chamber is subjected to an acoustic wave through the piezoelectric actuator, which causes a change in volume and, again, ejection of a drop of bioink through the nozzle [50].

The bioink material used with this printer should be viscous enough not to clog the nozzle, yet rigid enough that can withstand different mechanical stresses. One way to accomplish this is leaving the polymerization of the bioink to occur after it is deposited by the nozzle, which can negatively affect the fidelity of the construct [51]. Alternatively, a bioink with shear-thinning properties can be obtained, as in incorporating gelatin or collagen into the bioink [52]. Both inkjet printers have the advantage of the ability to create gradients of cell, material and growth factors by altering the size and shape of the droplet. A multi-nozzle printer has been developed to speed up the printing process and enable the printing of larger scaffolds [53].

Scientists have successfully printed neural, skin and cardiac tissue using the inkjet printer [50, 54, 55]. Regarding bone tissue engineering, 3D printing scaffolds for long bone reconstruction fabricated from different biomaterials proved to be effective [56]. Gao et al. fabricated bone tissue using the inkjet 3D bioprinting technology with polyethylene glycol dimethacrylate bioink and combined it with bone marrow-derived mesenchymal stem cells (BM-MSC). They further added either hydroxyapatite (Hap) or bioactive glass (BG) nanoparticles to compare their difference in enhancing the osteogenic potential of the bioink material. They found that fabricated scaffold with (Hap) had more osteogenic potential measured by alkaline phosphatase activity than scaffolds fabricated with the addition of BG [57]. Inzana et al. used inkjet printer to fabricate scaffold from calcium phosphate and implanted them on created femoral defect on animal models. They followed-up the defect to assess the healing and implants were shown to have an osteoconductive capacity with the ability of being self-degrading without adverse effect [58]. In human clinical trials, Saijo et al. fabricated scaffold from TCP powder using inkjet printer and implanted them in ten patients suffering from different maxillofacial defects. They reported the successful results ranged from the decrease in the operation time, the efficacy of the scaffold and its union with the surrounding bone and the negative overall adverse effect on these patients [59]. A Chinese scientist team also published their clinical trial report of using 3D-bioprinted titanium scaffold for treating different maxillofacial bone defects and mandibular osteotomy. Again, they reported shorter operation time and good aesthetic results [60].

### Extrusion bioprinting or filament printing

Like the inkjet printer, the bioink is stored in a chamber in the printer head, but, unlike the inkjet, a continuous filament of material is deposited on the platform and over a material layer to form the scaffold. It enables the use of highly viscous bioink that contains high cell density. It uses a dispenser system having a pneumatic, piston or screw and valve that creates large pressure on cells during extrusion. To save the viability of encapsulated cells, hydrogel is used to create a shear-thinning effect and allows easier passage of the cells through the nozzle and into the platform [61]. Saving the cell viability by adding the hydrogel affects the resolution of the constructed scaffold; thus, carefully choosing the bioink and its viscosity along with the nozzle diameter is needed to achieve the specific shape and size [62]. Many natural and synthetic bioinks having different properties had been tested for scaffold fabrication using the micro-extrusion printer [63].

Micro-extrusion printers have successfully printed cartilage, and scientists were able to develop ear and auricular cartilage using PCL mixed with cell-laden hydrogel [64]. Goh et al. used the same material PCL and the micro-extrusion technology to fabricate scaffolds to be placed on patient’s teeth extraction sites with the aim of preserving alveolar ridge height for later implant treatment [65]. They reported better preservation of alveolar height compared to patients who did not receive the PCL scaffold and caused a decrease in the alveolar ridge height.

Although micro-extrusion bioprinting is able to produce high cell density, it still carries the problem of the decreased resolution and lower cell viability hindering its translation.

### Laser-assisted bioprinting (LAB)

This is a nozzle-free technique; therefore, no nozzle clogging issue is encountered. The bioprinter is composed of a pulsated laser, a printer donor ribbon having two layers, an energy-absorbing layer and layer containing the bioink, which is heated by the laser and increases its pressure, causing a drop to be released. Lastly, there is a receiving substrate coated with hydrogel to cushion the falling drops of bioink and preserve its fidelity [66]. Some studies found that laser directly decrease the cell viability, but others demonstrated that an increase in the film thickness and viscosity of the bioink would rescue the cells and increase its viability [67, 68]. Gruene et al. studied the effect of using the ND:YAG-laser on the MSC viability laden in hydrogel used to construct 3D-bioprinted scaffold [69]. They found that laser caused an insignificant effect on the MSC’s viability and its ability to differentiate into osteocyte and chondrocyte.

Optimizing the physical parameters of this bioprinter and the bioink prove that laser bioprinting produces scaffolds with high resolution, as high as cell per drop. Unger et al. used time-resolved imaging to show the resolution of the scaffold by following the vapor bubble kinetics produced by the laser pulse until it causes the drop of bioink material into the collector slide [70]. Guillotin et al. defined the highest cell resolution bioprinting as producing a continuous line of cells in which cells are printed and placed one by one at the desired coordinate [71]. In their study, they deposited cells in rows utilizing laser bioprinter in a predefined location using computer-assisted geometric control with the highest precision and they also explained the dynamic and kinetics by which the bubble vapor and the produced bioink ejection occur. With the success of the 3D printing using the LAB, Michael et al. used it to fabricate skin by printing 20 layers of fibroblasts suspended in collagen above 20 layers of keratinocyte suspended in collagen and tested implantation of these construct on mice [72]. Mice had been followed-up for 11 days, and the constructs showed proper integration with the surrounding tissues.

In bone tissue engineering, Roskies et al. created defects on rabbit mandibles and reconstructed these defects using scaffolds fabricated from polyetherketoneketone (PEKK) combined with adipose-derived stem cell using LAB technology [73]. They followed their animals at 10-week and 20-week time, and they found proper integration of the implanted scaffold with the surrounding bone structure using CT scans and histological tests. Keriquel et al. moved a step ahead and developed a protocol using LAB for in situ bone bioprinting on mice to help precise positioning of cells for defect reconstruction [47]. The bone scaffold was fabricated from collagen and hydroxyapatite along with MSC to ensure its osteoconductivity. They tested the printing designs in vitro before applying and adapting the measurement to suit mice calvarial defects.

## Other factors for successful scaffold design

The success of the fabricated scaffold is markedly influenced by the bioink chosen and the 3D bioprinting chosen (Table 1) [48]. It also relies on other factors to guarantee the success of the scaffold, which are as follows:

**Table 1:** Bioprinting method and possible printable tissues

Inkjet bioprinting	Micro-extrusion bioprinting	Laser-assisted bioprinting
Aponeuroses Ear and nasal cartilage Mimetic muscles Blood vessels	Load-bearing bone Cartilage Muscle Nerves	Skin Blood vessels

### Pore size

The scaffold should interconnect pores to allow cell and nutrient infiltration and removal of waste [74]. Pore size further affects the cell migration and attachment along the scaffold to replace it. Very small pore size hinders the removal of waste and diffusion of nutrients while bigger pore size hinders intercellular ligand formation needed for cell migration. For bone tissue engineering, a pore size ranging from 100 μm to sometimes greater than 300 μm is needed as it provides the hypoxic condition that enhances both osteogenesis and angiogenesis for proper bone formation [75]. Liu et al. developed a sequential seeding protocol on 3D-bioprinted natural and synthetic scaffolds with human smooth muscle cells followed by umbilical vein endothelial cells (both are primary cell types for blood vessels), and with the aid of a bioreactor to ensure proper diffusion into the scaffold [76]. Their method enhanced vascularization of the 3D-printed scaffold as the presence of the smooth muscle cells enhanced the attachment of the endothelial cells, which can help forming a clinical-size scaffold.

Di Luca et al. investigated variating the scaffold pore sizes to achieve the gradient of oxygen needed, more in the peripheries and hypoxic at the center, and their influence on MSC differentiation [77]. The same hypothesis was also tested by Abbasi et al. and both studies concluded that using the gradient in pore size has a favorable effect on bone tissue engineering [78]. Controlling the porosity is achieved using the high control of the 3D printing and it also allows the formation of gradients that was hard to achieve using other scaffold techniques [79].

### Surface topography

Scaffold surface topography is found to have a direct effect on the MSC differentiation lineage and deciding its fate. A study conducted by Grazianio et al. in which they compared the use of smooth surface titanium scaffold, concave textured PLGA scaffold and convex textured of Hap scaffolds. They cultured MSC extracted from dental pulp (DP-derived MSC), and after 30 days, they measured alkaline phosphatase activity, detected bone proteins (osteocalcin and osteonectin) and observed cell under SEM [80]. They found that concave surfaces allowed for the most MSC differentiation into osteoblast and new bone tissue formation, followed by the smooth surface while, the least was found on the convex surface. The other study was undertaken by Shen et al., where they used the 3D printer to fabricate linear and wavy PCL scaffold and found that wavy scaffold design had higher osteogenic potential decided by having calcium deposition, increase alkaline phosphatase activity and osteocalcin staining [81]. The preference of the MSC for concave surfaces could be explained by how concavities provide a closer surface for cells allowing them to clump-forming denser integrin cell adhesion. Integrin is a molecule known to have a different effect on the cell; mostly related here to the differentiation of the MSC is integrin’s importance of signal transduction and passing cue from outside to the nucleus causing osteogenic gene expression [82].

## Conclusion

Scaffold fabrication for bone tissue engineering depends on many physical and chemical factors that cue simultaneously to ensure cell migration, proliferation, vascularization, osteogenic differentiation and scaffold degradation [83]. 3D bioprinting has proved to create the interconnected pores and the surface topography needed for bone tissue engineering, thereby overcoming one of the main challenges and barriers that faced the translation of technology [84]. Biomaterial combination, as well as methods and techniques for every organ, still need to be tested and assessed prior to their translation into patient care [85]. ClinicalTrials.gov lists only one clinical trial investigating the safety and efficacy of treating bone injuries using 3D scaffolds made from demineralized bone matrix and seeded with bone marrow-derived stem cells [86]. Apart from the bone tissue engineering, several organs have been successfully engineered and successfully transplanted into patients, such as the trachea, urethra and blood vessels [87, 88].

One of the barriers that hinders the translation of the technology is its high cost. However, once a bioprinter is owned, fabricating scaffolds using the 3D printing is relatively cost-effective as only the necessary material is used with minimal waste [89]. A bioengineering research group has overcome this barrier by describing a method to modify an off-the-shelf 3D printer into a printer for bio-fabrication use [90].

The technology of 3D printing is promising and allows for individualized medicine that is currently progressing. Researchers are continuing to improve and develop the deficits seen with tissue 3D bioprinting. Ultimately, 3D printing technology is likely to become, not far from now, an essential tool for maxillofacial, plastic and reconstructive surgeons potentially to improve facial reconstruction surgical outcomes, along with patient satisfaction and the quality of life of patients.

## Data Availability

Not applicable.

## Abbreviations

3D bioprinting: Three-dimensional bioprinting
AM: Additive manufacturing
BG: Bioactive glass
BMP: Bone morphogenic protein
CAD/CAM: Computer-aided design/computer-aided manufacturing
ECM: Extracellular matrix
FGF: Fibroblast growth factor
Hap: Hydroxyapatite
IGF: Insulin-like growth factor
LAB: Laser-assisted bioprinting
MSC: Mesenchymal stem cell
PCL: Polycaprolactone
PGA: Polyglycolic acid
PLA: Polylactic acid
PLGA: Polylactic-glycolic acid
SCPL: Solvent casting and particulate leaching
VEGF: Vascular epithelial growth factor
